# Unannotated Open Reading Frame in *Saccharomyces cerevisiae* Encodes Protein Localizing to the Endoplasmic Reticulum

**DOI:** 10.17912/micropub.biology.000992

**Published:** 2023-10-20

**Authors:** Scott Chang, Matthew Joyson, Anna Kelly, Lucas Tang, John Iannotta, April Rich, Nelson Castilho Coelho, Anne-Ruxandra Carvunis

**Affiliations:** 1 Computational and Systems Biology, University of Pittsburgh School of Medicine; 2 Pittsburgh Center for Evolutionary Biology and Medicine; 3 Joint CMU-Pitt PhD Program in Computational Biology

## Abstract

There are thousands of unannotated translated open reading frames (ORFs) in the *Saccharomyces cerevisiae* genome. Previous investigation into one such unannotated ORF, which was systemically labeled YGR016C-A based on its genomic coordinates, showed that replacing the ORF’s ATG start codon with AAG led to a change in cellular fitness under different stress conditions (Wacholder et al., 2023). This suggested translation of YGR016C-A plays a role in cellular fitness. Here, we investigate Ygr016c-a’s subcellular localization to gain insight into its cellular function. Computational prediction tools, co-expression analysis and fluorescence microscopy suggest that the Ygr016c-a protein localizes to the endoplasmic reticulum.

**Figure 1. Ygr016c-a protein localizes to the endoplasmic reticulum:**
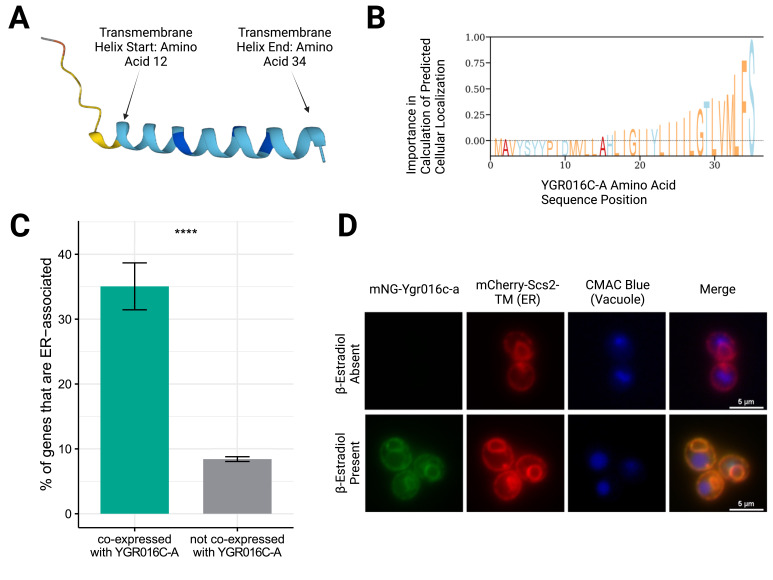
(A) The protein structure for Ygr016c-a was predicted using ESMFold (Lin et al., 2022). The colors (orange, yellow, light blue, and dark blue) represent predicted-LDDT confidence intervals for the modelled residues (<0.5, 0.5-0.7, 0.7-0.9, and 0.9-1.0 confidence, respectively). From amino acid position 11-34, an alpha helix is predicted with a confidence of at least 0.7. Ygr016c-a is predicted to possess a transmembrane domain from amino acid position 12-34 using Phobius (Käll et al., 2004, 2007), which is the basis for the transmembrane helix Start and End annotations on the structure prediction. (B) The DeepLoc 2.0 prediction tool (Thumuluri et al., 2022) reported Ygr016c-a to localize to the endoplasmic reticulum (ER) with a probability of 0.7518, which is higher than the background threshold of 0.6090. Represented here is the emphasis DeepLoc placed on each residue, with higher importance on the residues toward the C-terminus. (C) Analysis of co-expression data curated by Rich et al. (2023) compares the percentage of genes co-expressed with YGR016C-A whose encoded proteins localize to the ER (n=61 ER-localizing, 35.1% of 174 total co-expressed; green bar) to the percentage of genes not co-expressed with YGR016C-A whose encoded proteins localize to the ER (n=483 ER-localizing, 8.4% of 5734 total non-co-expressed; gray bar). The error bars represent the standard error of the percentage (see methods). The four stars (****) represent the high significance conferred by a p-value of 2.2e-16, calculated with Fisher’s exact test. (D) A yeast strain containing the construct *Z _3_ EVpr-mNG-YGR016C-A:HYG* at the *HIS3* locus was imaged on an epifluorescence microscope without (top row) and with (bottom row) induction by β-estradiol. The first column (left to right) shows the localization of the fusion protein mNG-Ygr016c-a, while the second column represents the localization of the ER marker, Scs2TM fused with mCherry. The CMAC blue dye was used to dye the vacuole as seen in the third column. The last column shows the merge of all channels, confirming the co-localization of the fused protein mNG-Ygr016c-a with the mCherry-Scs2TM ER marker. Cells shown are representative of the entire field.

## Description

The *Saccharomyces cerevisiae* genome contains approximately 6,000 annotated open reading frames (ORFs) that are translated into proteins (Goffeau et al., 1996) . Recently, a large-scale meta-analysis of ribosome profiling results demonstrated that in addition to these annotated ORFs, there are 18,953 unannotated ORFs that are also translated (Wacholder et al., 2023) . A subset of this group of ORFs was further studied, with some showing experimental evidence of affecting cellular phenotype and fitness (Wacholder et al., 2023) . Among the unannotated translated ORFs exhibiting cellular effects in deletion screenings is an ORF that encodes for a 35 amino acid microprotein of unknown function (Wacholder et al., 2023) which was given the systematic name YGR016C-A based on its genomic coordinates, chrVII:523246-523353(-). This unannotated ORF was shown to cause a change in cell fitness under different stress conditions when its normal ATG start codon was mutated to an AAG (Wacholder et al., 2023) . This suggested translation of YGR016C-A plays a role in cellular phenotype and fitness. To further characterize this ORF, we combined computational prediction tools and fluorescence microscopy to determine the cellular localization of Ygr016c-a.

ESMFold, a deep learning model that has demonstrated high levels of accuracy pertaining to protein structure prediction based on amino acid sequences, was used to predict structure (Lin et al., 2022) . ESMFold predicted that Ygr016c-a would exhibit a large α-helix between residues 11 to 34 with a confidence score of at least 0.7 ( Figure 1A ). Next, Phobius (Käll et al., 2004, 2007) *,* a computational tool used for predicting the probability of transmembrane domains for a given amino acid sequence, reported Ygr016c-a to have a singular transmembrane helix ( Figure 1A ) between amino acids 12-34, which aligns with the α-helix reported by ESMFold. Since transmembrane helices interact with either the plasma membrane or the membranes of specific organelles, this result suggests a non-cytoplasmic localization of Ygr016c-a. To predict the localization of Ygr016c-a, DeepLoc 2.0 (Thumuluri et al., 2022) , a protein language model relying on amino-acid sequence input, was used. Notably, this tool outputs the probability of the protein localizing to distinct subcellular localizations and a corresponding threshold probability for each localization. If the calculated probability of a protein’s localization is less than the threshold probability, the probability is considered not significant. The only localization where Ygr016c-a had a calculated probability of localization (0.752) above the threshold (0.609) was the endoplasmic reticulum (ER; Figure 1B ). Additionally, DeepLoc placed higher importance on residues towards the C-terminus when calculating this localization ( Figure 1B ). Gene co-expression networks can be used for function and localization inferences (van Dam et al., 2018) . Considering the predicted ER membrane localization, we checked to see if genes that are known to be associated with the ER have similar transcript expression patterns as YGR016C-A using co-expression data (Rich et al., 2023) and cellular component annotations from the Gene Ontology (GO) database (Ashburner et al., 2000; The Gene Ontology Consortium et al., 2023) . 35.1% of genes co-expressed with YGR016C-A localize to the ER, while only 8.4% of genes that aren’t co-expressed with YGR016C-A localize to the ER ( Figure 1C ). We calculated that annotated ORFs that are co-expressed with YGR016C-A are 5.9 times more likely to have an ‘ER’ GO cellular component annotation than genes that are not co-expressed with YGR016C-A (Odds Ratio, 95% confidence interval: 4.2-8.2, p-value < 2.2e-16, Fisher’s Exact test; Figure 1C ). Holistically, these computational analyses provided evidence that Ygr016c-a may localize to the ER.

Wacholder et al., 2023 tagged YGR016C-A at the endogenous locus under its native promoter with a fluorescent protein at the C-terminus and reported inconclusive localization, likely due to low native expression levels. In our study, to test our computational predictions, we genetically engineered a *S. cerevisiae* strain to overexpress the ORF YGR016C-A fused with a mNeonGreen (mNG) tag at the N-terminus. This overexpression was driven by the Z _3_ EV promoter, a β *-* estradiol inducible promoter (Ohira et. al, 2017) . This construct was integrated at the *HIS3* locus. Furthermore, these cells were also transformed with a plasmid expressing mCherry-Scs2TM, a well-known ER marker (Zhou et al., 2014) . Fluorescence microscopy showed co-localization of the mNG-Ygr016c-a fusion and the mCherry-Scs2TM fusion ( Figure 1D ), confirming the predicted ER localization for Ygr016c-a.

Localization of a protein can provide insight into its potential function (Pan et al., 2021) . Here, we have shown ER localization and a predicted C-terminal transmembrane helix for Ygr016c-a. A protein with a lone transmembrane domain at the C-terminus of the peptide chain is considered a tail anchored protein (Schuldiner et al., 2008). The Guided Entry of Tail-anchored proteins (GET) pathway transports tail anchored proteins to the ER (Schuldiner et al., 2008). Therefore, Ygr016c-a may localize to the ER via the GET pathway. This is a possible avenue for future research.

## Methods

Sequence and chromosome location of unannotated ORF known as YGR016C-A

The name YGR016C-A given to the unannotated ORF was chosen using the naming system based on SGD conventions.

The sequence of YGR016C-A is: ATGGCGGTTTATTCATACTATCCAATTGACATGGTTTTGCTCGCTCACCTCATTGGCATCATCTACTTAATTATAATTCTAGGTACATTGGTCATGTTGTTTTCTTGA

Furthermore, the ORF is located on the minus strand of chromosome VII between coordinates 523246 and 523353.

Structure, TM and localization predictions

The protein structure of Ygr016c-a was predicted by inputting the amino acid sequence to the online version of ESMFold (Lin et al., 2022) . The website was accessed at: https://esmatlas.com/resources?action=fold on 26 May 2023.

The prediction of transmembrane helices was computed by inputting the amino acid sequence to the online version of Phobius (Käll et al., 2004, 2007). The website was accessed at: https://phobius.sbc.su.se/ on 11 August 2023.

The predicted cellular localization was determined by inputting the amino acid sequence to the online version of DeepLoc 2.0 (Thumuluri et al., 2022) . The website was accessed at: https://services.healthtech.dtu.dk/services/DeepLoc-2.0/ on 29 May 2023.

Co-expression and ER enrichment analysis

Co-expressed was defined as a value greater than the 99th percentile for all co-expression values in the Rich et al. coexpression data; i.e., 99th percentile of all pairwise combinations for the 11,630 ORFs in the Rich et al. co-expression data (co-expression value > 0.836). The coexpression matrix was then subdivided to only include genes that have at least one GO cellular component annotation using the GO slim annotation file downloaded from SGD on 20 January 2021 (Saccharomyces Genome Database). This resulted in n = 5,908 genes. Of this subset, 174 genes were co-expressed with YGR016C-A and 5,734 genes were not co-expressed with YGR016C-A. Any gene with at least one GO cellular compartment annotation with the words ‘endoplasmic reticulum’ was labeled as ‘ER’ associated (n= 544) and all other genes were labeled as ‘not ER’ associated (n= 5,364). Fisher’s exact test was subsequently used to quantify the significance of enrichment using the R function fisher.test(). Standard error of the percentage was calculated using the following equation: root-square (percentage*(100-percentage)/n)

*Saccharomyces cerevisiae* strain

2x Master mix Q5 Hot Start polymerase (New England BioLabs) was used to amplify the construct containing Z _3_ EVpr-mNG-YGR016C-A:HYG, using plasmid pARC0400 as template. Plasmid pARC0400 was made by LR recombinase (ThermoFisher) between an Entry Clone containing the YGR016C-A ORF and a destination plasmid containing Z _3_ EVpr-mNG-ccdB:HYG. The PCR product containing homology to *HIS3* locus was used to transform the strain yARC0085 following the LiAc/PEG/ssDNA transformation protocol (Dunham, 2015) . Positive transformants were selected on YPD+Hygromycin (200μg/ml) and used for a subsequent transformation with the plasmid pARC0006 (containing the mCherry-SCS2TM). The final transformants were selected on SC-LEU+GLU+Hygromycin (200μg/ml).

Microscopy methods

Yeast cells were inoculated into overnight cultures of SC-LEU+GLU+Hygromycin. The following day the culture was diluted to an optical density of 0.2 at 600nm, and after 1 hour of growth at 30°C with constant shaking at 120rpm, half of the culture was inoculated with β-estradiol (to a final concentration of 10μM) to induce the expression of mNG-YGR016C-A. In parallel, 100% ethanol was added to the other half of the culture to serve as negative control for the induction.

After 3 hours of induction, the vacuolar dye CMAC Blue (ThermoFisher) was added to the cells to a final concentration of 1μM and incubated at room temperature for 15-minutes prior to imaging. Cells were transferred to glass bottom culture dishes and imaged on a NIKON Ts2R-FL Epifluorescence microscope (Nikon) with a camera ORCA-Flash 4.0. All images were taken using 100x oil immersion objective and analyzed with the software NIS Elements (Nikon).

## Reagents

**Table 1 | Plasmids, strains and reagents used in this study**

**Table:** 

**Plasmids**				
**Name**	**Insert**	**Description**		**Source**
**pARC0006**	*mCherry-SCS2TM*	Plasmid containing *mCherry-SCS2TM* . *LEU2* gene for yeast selection.		**Zhou et al. 2014**
**pARC0400**	* Z _3_ EVpr-mNG- YGR016C-A:HYG *	Expression plasmid used to amplify the construct *Z _3_ EV-mNG-YGR016C-A:HYG* to integrate at *HIS3* locus		**This study**
**Strains**				
**Name**	Genotype			**Source**
**yARC0085**	*MAT* α *ura3Δ0* *leu2Δ* ::ACT1pr-Z _3_ EV:NatMX			**McIsaac et al. 2013**
**yARC0988**	*MAT* α *ura3Δ0* *leu2Δ* ::ACT1pr-Z _3_ EV:NaTMX *his3Δ* : Z _3_ EVpr-mNG-YGR016C-A:HYG + plasmid pARC0006 (mCherry-SCS2TM)			**This study**
**Reagents**				
**Name**	**Final Concentration**	**Reference**		**Company**
**2x Q5 Master Mix**	1x	M0492L		**NEB BioLabs**
**LiAc**	10mM	L4158		**Millipore Sigma**
**PEG**	37%	P4338		**Millipore Sigma**
**ssDNA**	2mg/ml	15632-011		**Invitrogen**
**Hygromycin B**	200μg/ml	H75020		**Research Products International**
**CMAC Blue**	1μM	C2110		**ThermoFisher**
β **-estradiol**	10μM	E8875		**Millipore Sigma**
**Primers**				
**Name**			**Sequence**	
***HIS3* integration primer Fw **			5’-TCTATATTTTTTTATGCCTCGGTAATGATTTTCATTTTTTTTTTTCCACCTAGCGGATGACTCTTTTTTTTTCTTAGCGATTGGCATTATCACATAATGAATTATACATTATATAAAGTAATGTGATTTCTTCGAAGAATATACTAAAAAATGAGCAGGCAAGATAAACGAAGGCAAAGacaaaagctggagctctagta-3’	
***HIS3* integration primer Rv **			5’-AAAGAAAAAGCAAAAAGAAAAAAGGAAAGCGCGCCTCGTTCAGAATGACACGTATAGAATGATGCATTACCTTGTCATCTTCAGTATCATACTGTTCGTATACATACTTACTGACATTCATAGGTATACATATATACACATGTATATATATCGTATGCTGCAGCTTTAAATAATCGGTGTCAgcgaattgggtaccggcc-3’	
